# Men and Those With a History of Smoking Are Associated With the Development of Postoperative Ileus Following Elective Colorectal Cancer Resection at a Private Academic Hospital in Johannesburg, South Africa: A Retrospective Cohort Study

**DOI:** 10.3389/fsurg.2021.667124

**Published:** 2021-06-15

**Authors:** Estella L. Watkins, Natalie Schellack, Veena Abraham, Brendan Bebington

**Affiliations:** ^1^Mediclinic Southern Africa, Wits Donald Gordon Medical Centre, Johannesburg, South Africa; ^2^Department of Pharmacology, Faculty of Health Sciences, University of Pretoria, Pretoria, South Africa; ^3^School of Pharmacy, Sefako Makgatho Health Sciences University, Garankuwa, South Africa; ^4^Department of Surgery, Faculty of Health Sciences, University of the Witwatersrand, Johannesburg, South Africa

**Keywords:** colorectal cancer resection, preoperative risk assessment, postoperative ileus, male gender, smoking

## Abstract

**Introduction:** A scarcity of local published data on colorectal cancer (CRC) postoperative complications, including postoperative ileus (POI), exists. POI is a temporary gastrointestinal (GI) state of absent or reduced gastric motility shown to increase patient morbidity, prolong length-of-stay (LOS), and intensify the healthcare resource burden. The pathogenesis of POI involves a neurogenic and inflammatory phase plus a pharmacological component.

**Aim and Objectives:** This study aimed to determine centre-specific preoperative risk factors associated with the development of ileus post elective therapeutic CRC resection. The objectives were to determine whether patient demographics; functional status; comorbidities; GI history; pharmacotherapy (including neoadjuvant chemotherapy); and lastly neoadjuvant radiation and chemoradiation were associated with the development of POI.

**Method:** Patients who underwent CRC resection between January 2016 and May 2019 were retrospectively identified from an existing database. Urgent—or non-therapeutic surgeries; surgeries with the complication anastomotic leak or GI obstruction; patients under 18 at the time of surgery or surgeries preceded by preoperative parenteral nutrition were excluded. A comparison was done of the incidence of exposure in the study cohort to investigated variables as potential risk factors for the complication POI.

**Results:** A total of 155 patient cases were included, and 56 (36%) of them developed POI. Univariate comparison of patients who developed POI with demographic characteristics of patients who did not suggested that women were at lower risk to develop POI compared to men (*p* = 0,013; RR 0,56; 95% CI 0,36–0,89). Functional status suggested that all previous smokers were at a higher risk to develop POI compared to lifetime non-smokers (*p* = 0,0069; RR 1,78; 95% CI 1,17–2,70). Multivariable comparison of ≤ 5 qualifying parameters showed no significance.

**Conclusion:** The high local incidence of POI in this patient population shows that intervention is required to reduce the POI rate and improve postoperative outcomes. This study suggests that for men and all patients with a history of smoking both, CRC resection preoperative recommendations with the intention to prevent POI should include instructions initiating the activation of preventive strategies like the Enhanced Recovery After Surgery (ERAS) programme. More studies are needed to adequately determine local perioperative risk factors for POI.

## Introduction

Colorectal cancer (CRC) falls under the top five most widespread cancers in both genders in South Africa (SA) (1, 2). To treat CRC a multimodal multidisciplinary team approach is recommended, but the surgical resection of the principal lesion continues to be the mainstay of treatment to accomplish cure or to extend the life expectancy of the patient (2, 3). Postoperative ileus (POI) is a common complication following CRC resection (4, 5). In one large study looking at the association of specific operative complications with outcomes post elective colon resection, ileus was found to be the most common index complication as compared to (listed from most common to least): bleeding; incisional surgical site infection; anastomotic leak; urinary tract infection; venous thromboembolism; pneumonia; and myocardial infarction (6). POI incidence may range between 2 and 54%, with the median international incidence reported as 10,3% (5). POI describes a temporary gastrointestinal (GI) state of absent or reduced gastric motility or “peristalsis” following surgical intervention and is attributable to non-mechanical causes (7). During POI, when peristalsis fails, GI secretions build up, leading to abdominal distension, nausea and vomiting, and delayed passage of flatus and stool (8). POI may require the placement of a nasogastric tube or even parenteral nutrition (9). POI slows recovery—prolonging hospital stay, it also increases postoperative morbidity, healthcare provider costs and intensifies the healthcare resource burden (7, 10, 11). POI after colorectal surgery has been shown to also increase the 30–day unplanned patient readmission rates (12). Patients who develop POI are usually more dissatisfied with the surgical outcome after suffering increased anxiety, abdominal pain and discomfort, and decreased mobility (7, 13).

The pathogenesis of POI typically involves the consolidation of three components, the early transient neurogenic phase (ending within 3 h after surgery), the subsequent inflammatory phase (starts 3–4 h after surgery but continues for much longer than the neurogenic phase), and a further reduction in gastric motility caused by exogenic pharmacological substances (14). When POI after GI surgery continues beyond the expected duration with the radiological exclusion of small bowel obstruction, “paralytic” or prolonged POI (PPOI) is diagnosed (8, 15). For the purpose of this study, the term POI will be used interchangeably with the term PPOI.

Certain patients may be predisposed to developing POI due to patient demographics or variables including increasing age (10); being men (11, 16–19); chronic preoperative opioid use (13); neoadjuvant chemotherapy (18); and previous abdominal surgery (13), amongst other reasons, all of which are usually known at the time of surgery (7). Current best practise management of POI in Europe and Australasia still lacks robust evidence (5). Supportive care of POI includes non-routine placement of a nasogastric tube to relieve refractory nausea and vomiting with abdominal distension and parenteral nutrition to prevent compromised postoperative nutrition when the patient is unable to tolerate an oral diet (7). Management is mostly still conservative and considered experimental making it a necessity to focus on preventative strategies once centre-specific perioperative variables associated with POI are known (11). Strongly recommended preventative strategies for POI include the implementation of an Enhanced Recovery After Surgery (ERAS) programme (19).

This study aimed to determine centre-specific preoperative risk factors associated with the development of POI as a complication of post-elective therapeutic resection for CRC. To date, there is a scarcity of published data in SA on presentation and outcomes of patients with colorectal disease, especially CRC (20). The recent publication “A 1 year audit of the Colorectal Unit at Wits Donald Gordon Medical Centre: 2016–2017” by Lutrin et al. was one of the first publications of its kind in SA (20). In this publication, the activity of the Wits Donald Gordon Medical Centre (WDGMC) Colorectal Unit over a 12-month period was assessed. Variables like surgical indication and length of stay (LOS) as well as post-elective colorectal resection complication rates, including the rate of POI at WDGMC, were reported. Perioperative risk factors for POI or any other postoperative complication were not determined. The objectives of the present study were to determine whether patient demographics; preoperative functional status; comorbidities at the time of surgery; GI history and preoperative pharmacotherapy, including neoadjuvant chemotherapy and lastly radiation therapy and chemoradiation were associated with the development of POI. The rationale of this study was to investigate local preoperative risk factors at the study hospital for the development of POI to help identify high-risk patients before the therapeutic surgery, which should help to increase compliance to principles of preventative POI strategies like ERAS.

## Materials and Methods

### Study Site

This retrospective cohort study was conducted at a Private Academic Hospital in Parktown, Johannesburg, Gauteng Province, SA. At the time of data collection, the hospital had 190 beds and several highly specialised units, including a Colorectal Unit. The Colorectal Unit at the study hospital is a tertiary referral centre led by a group of colorectal surgeons and offers comprehensive care to patients presenting with all types of colorectal disorders including CRC. A diverse team of healthcare professionals ensures a multimodal team approach.

### Study Design

A retrospective cohort study design was followed. Permission was granted to access the South African Medical Research Council (SAMRC) CRC study Research Electronic Data Capture (REDCap) database used to record variables for adult patients with CRC for a previous multidisciplinary, longitudinal cohort study (21). Relevant data of patients recruited specifically at the study hospital were retrieved and further supplemented with preoperative assessment data obtained from the Preoperative Assessment Clinic at the study hospital.

### Study Population and Sample Selection

All cases of patients were those of 18 years of age or older who underwent elective therapeutic resection for CRC at the study hospital from the foundation of the SAMRC CRC study REDCap database in January 2016 to the date of Wits Human Research Ethics Committee (HREC) (Medical) Ethics clearance, May 31, 2019, formed the study population that was considered for inclusion into this study according to the inclusion and exclusion criteria of the study. Surgical cases were excluded if the patient was younger than 18 years old at the time of the therapeutic surgery; if the surgery was non-elective (urgent or emergency surgery) or a pre-therapeutic surgery or secondary surgery for a complication; if the patient developed GI obstruction or had an anastomotic leak/ breakdown after the resection; if the patient received preoperative total parenteral nutrition (TPN) or if the therapeutic resection occurred prior to the foundation or launch of the SAMRC CRC database or after the Wits HREC approval date.

### Data Collection and Data Collection Instrument

The data collection instrument was designed based on the following references: (4, 10, 11, 13, 15–18, 22–25). The REDCap platform was hosted and managed by the University of Witwatersrand, Johannesburg, SA (26–28). POI was clinically diagnosed according to the American College of Surgeons- National Surgery Quality Improvement Program (ACS-NSQIP) definition: “any NGT use or NPO status on postoperative day 4 or later” (18) with the radiological exclusion of small bowel obstruction, and documented by the examining colorectal surgeon prior to being entered under postoperative complications on the SAMRC CRC study REDCap database. The following definitions were used during the data collection: unexplained weight loss: if you lose more than 5% of your body weight over 6 months to a year without trying to lose weight (29). Underweight: BMI < 18,50 kg/m^2^; normal weight range: BMI 18,50–24,99 kg/m^2^; overweight: BMI ≥ 25,00 kg/m^2^; obese: BMI ≥ 30,00 kg/m^2^ (30). Smoking and alcohol use was recorded as current if the patient is currently still using, or if they stopped <5 years ago. If they stopped more than 5 years ago, it was marked as previous (as per standard clinical practise at the study hospital). Chronic use definition: the use of opioids or steroids on a frequent basis as recorded during the preoperative assessment no more than 180 days before the time of admission. Chronic medication definition: pharmacotherapy used on a frequent basis for chronic diseases as listed by the Council for Medical Schemes (24).

Data on the following modules were collected:

A. Patient demographics (age at the time of surgery; gender; ethnicity; tumour stage; location of malignancy; surgical procedure (first/second, urgency (for inclusion/exclusion criteria); procedure; complications existing at the time of surgery; access; stoma formation).

B. Functional status [BMI within 42 days before the surgery (31)—to classify the patient as underweight, normal weight range, overweight and obese (24); smoking status; alcohol use; ASA grading within 42 days before the surgery; NYHA classification within 42 days before the surgery].

C. Modified Charlson comorbidity index (modified CCI) score from comorbidity data within 42 days before the surgery; hypertension.

D. GI medical history [duration of symptoms prior to diagnosis; previous GI diagnosis (if Inflammatory bowel disease (IBD): type); previous abdominal surgery].

E. Pharmacotherapy: all acute medication taken in the last 180 days before the surgery and all chronic medication, regardless of when it was recorded in relation to the surgery. All neoadjuvant chemotherapy received before the surgery (due to chronic chemotherapy-induced constipation (CIC) (25, 32) (location of malignancy; status; intent; type; number of cycles; complications (and type, outcomes); completion status) and preoperative administration of monoclonal antibodies before the surgery.

F. Neoadjuvant radiation within 180 days before the surgery and all chemoradiation regardless of when it was administered before the surgery [location of malignancy; intent; sensitising agent (and type); complications (and type, outcomes); and completion status].

### Statistical Analysis

The sample size of 155 cases (56 with POI; 36%) allowed the estimation of Relative Risks of 1.45 or greater with 80% power at the 5% significance level, which is adequate for a study of this nature (33). Descriptive analysis of the data was carried out as follows: categorical variables were summarised by frequency and percentage tabulation, and some were illustrated using bar charts. Continuous variables were summarised by the mean, standard deviation, median and interquartile range, and their distribution illustrated by histograms. The Relative Risk of each study variable for the development of POI was determined, together with its 95% confidence interval (CI), using binomial regression. Categories with *n* < 15 overall were not included in analyses (no reliable inference could be made based on such small groups). Study variables significant at *p* < 0.20 were combined into a multivariable model, after examining each pair of variables for possible confounding using the chi-squared test (or Fisher's exact test for 2 × 2 tables) (34). A value of Cramer's V (or the phi coefficient for Fisher's exact test) > 0.50 was regarded as too strong an association to include both variables in the multivariable model. Data analysis was carried out using SAS version 9.4 for Windows. The 5% significance level was used.

## Results

**Figure 1:**
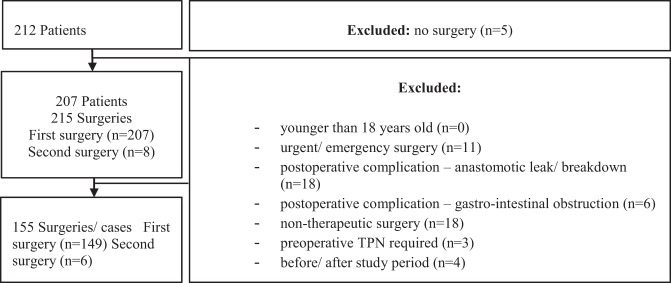
Exclusions made from REDCap database.

As illustrated by Figure 1, the study population comprised 212 patients. For 5 patients, a non-surgical approach was followed, and the other 207 patients underwent a collective amount of 215 surgeries. After the exclusion criteria were applied, descriptive and comparative data analysis proceeded with the data collected on the remaining 155 elective therapeutic surgeries (refer to Appendix: Supplementary Data, Table 1 in Supplementary Material).

### Module A—Patient Demographics

As seen in Table 1 below: most patients (34,2%) were between 60 and 69 years of age at the date of surgery (20,6% 18–49; 25,8% 50–59; 19,4% 70 + years of age).

**Table 1:** Patient demographics.

**Variable**	**Category**	**Overall**	
		***n*** **=** **155**	
		***n***	**%**
**Module A—patient demographics**			
Age at time of surgery	18–49y	32	20,6
	50–59y	40	25,8
	60–69y	53	34,2
	70y+	30	19,4
Gender	Man	81	52,3
	Woman	74	47,7
Ethnicity	White	108	69,7
	Black	20	12,9
	Indian	19	12,3
	Other	8	5,2
Tumour stage	Local	65	41,9
	Regional	61	39,4
	Distant	29	18,7
Location of malignancy	Colon (right) No	131	84,5
	Colon (right) Yes	24	15,5
	Colon (left) No	113	72,9
	Colon (left) Yes	42	27,1
	Colon (rectum) No	65	41,9
	Colon (rectum) Yes	90	58,1
	Synchronous lesion No	154	99,4
	Synchronous lesion Yes	1	0,6
Surgical procedure	Low anterior resection (LAR) (rectal)	81	52,3
	Right hemicolectomy (right sided)	22	14,2
	Abdominal perineal resection (APR) (rectal)	23	14,8
	Other (*n* < 15 per type of surgical procedure)	33	18,7
Surgical access	Open operation	93	60
	Laparoscopic completed	57	36,8
	Laparoscopic assisted open operation	3	1,9
	Laparoscopic converted to open	2	1,3
Stoma formation	No	56	36,1
	Yes	99	63,9
Cancer-related complication/s at surgery	No	123	79,4
	Yes	32	20,6
Postoperative	No	99	63,9
complication ileus	Yes	56	36,1

52,3% of the patients were men. The POI rate was 36% (56 POI out of 155 cases). The majority of the patients were white (69,7%). Most tumours were staged as either local (41,9%), or regional (39,4%). One patient had a synchronous lesion, so the presence/ absence of each location was considered separately.

The majority of tumours (58,1%) were located in the rectum. 27,1% were located in the left colon and 15,5% in the right colon. 52,3% of the patients had a lower anterior resection. The majority of patients (60,0%) had an open operation. 63,9% of the patients' operations included stoma formation. For 3,9% of the patients, this was their second therapeutic surgery (Figure 1), and 20,6% of the patients had cancer-related complications at the time of the elective surgery (see Table 1).

When patients who developed POI were compared with patients who did not develop POI, demographic characteristics suggested that women were at lower risk to develop POI compared to men (Appendix: *p* = 0,013; RR 0,56; 95% CI 0,36–0,89). There was no significant association between POI and the following variables: age at surgery; ethnicity; tumour stage; location of malignancy; surgical procedures with *n* > 15 [lower anterior resection (LAR); right hemicolectomy (right sided) and abdominal perineal resection (APR) (rectal)]; cancer-related complications at time of surgery; surgical access and stoma formation. The following variables were not analysed due to either high levels of missing data or categories with *n* < 15 at the overall level: synchronous lesion; procedures with *n* < 15; type of cancer-related complications existing at the time of surgery.

### Module B—Functional Status

**Figure 2:**
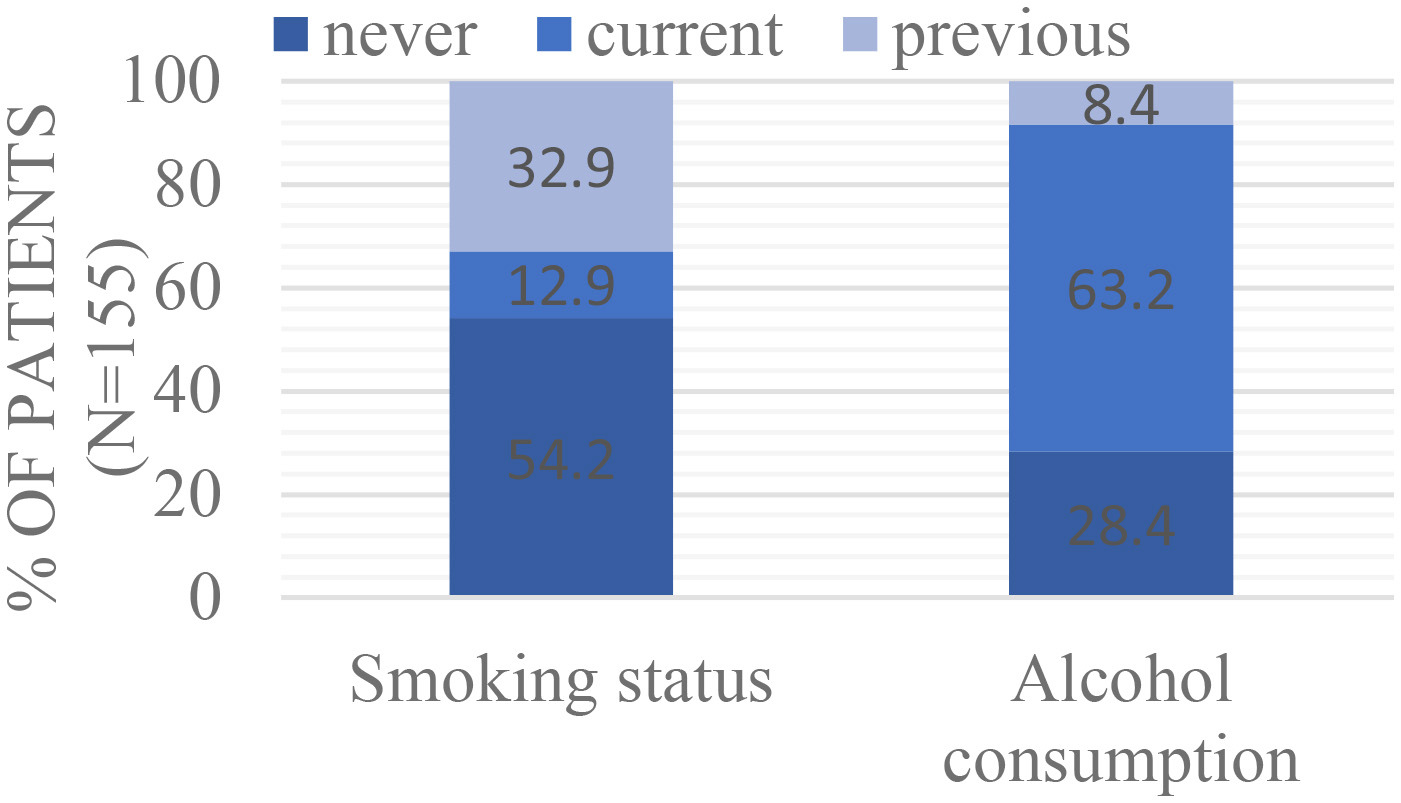
Smoking status and alcohol consumption.

64,3% of the surgeries were on patients with a BMI ≥ 25 (*n* = 129; 16,8% missing data). Smoking status and alcohol use (see Figure 2 below): 12,9% (*n* = 20) and 63,2% (*n* = 98) of the patients were current smokers and users of alcohol, respectively. 32,9% (*n* = 51) and 8,4% (*n* = 13) of the patients were previous smokers and users of alcohol respectively and 54,2% (*n* = 84) and 28,4% (*n* = 44) of patients never smoked or used alcohol.

When patients who developed POI were compared with patients who did not develop POI, functional status suggested that previous smokers were at a higher risk to develop POI compared to lifetime non-smokers (*p* = 0,0069; Table 2: RR 1,78; 95% CI 1,17–2,70). As seen in the Appendix, there was no significant association between POI and BMI; current smoking and alcohol use. ASA grading and NYHA classification was not analysed due to missing data that exceeds 30%.

**Table 2:** Known risk factors associated with POI.

**Patient demographics**	**Independent risk factors–references**	**Associated variables–references**
Age at time of surgery	(10) (elective colorectal surgery); (13) (laparoscopic colectomy); (15) (radical gastrectomy for cancer); (18) (elective colectomy)	–
Gender: man	(11) (colorectal resection), (16) (elective colorectal resection), (17) (colorectal cancer resection), (18)	(35) (major abdominal surgery)
Disseminated cancer	(36) (elective colon resection)	–
Tumor-node-metastasis (TNM) Stage III (gastric cancer)	(15)	–
Procedure type: right hemicolectomy, total colectomy and reversal of Hartmann's or end ileostomy	–	(16)
Procedure type: ileocolonic anastomosis	(36)	–
Procedure type: rectal resection	(11)	–
Open technique	(11, 16, 18, 35)	(15)
Conversion to laparotomy	(11)	–
Laparoscopic technique significantly associated with decreased prolonged ileus risk	(36)	–
Stoma formation	-	(16, 17)
**Functional status**	**Independent risk factors**	**Associated variables**
American society of anaesthesiologist (ASA) classification	–	(15, 16)
Age-adjusted charlson comorbidity index (ACCI)	4 (colorectal resection)	-
Smoker	(18, 37) (colon resection for diverticular disease)	–
Smoking history	(35)	–
Increased body mass index (BMI)	(18) (elective colon resection), (23)	(15)
**Comorbidities**	**Independent risk factors**	**Associated variables**
Chronic obstructive pulmonary disease (COPD)	(36)	–
Peripheral vascular disease (PVD) comorbidity	(17)	–
Respiratory comorbidity	(17)	–
**Gastrointestinal medical history**		
Previous abdominal operation	(13)	–
**Pharmacotherapy**	**Independent risk factors**	**Associated variables**
Preoperative chronic narcotic use	(13)	–
Neoadjuvant chemotherapy	(18)	–
Preoperative oral antibiotic bowel preparation associated with decreased prolonged ileus risk	(18, 36)	–
Lack of preoperative oral antibiotic bowel preparation	(18)	–

### Module C—Modified Charlson Comorbidity Index; Hypertension

**Figure 3:**
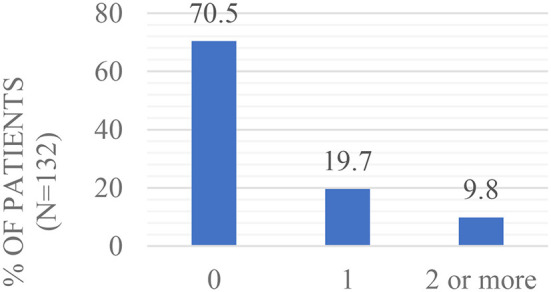
Modified CCI score.

The majority of patients had a CCI score of 0 (70,5%; *n* = 132), see Figure 3 below:

37,1% of the patients were hypertensive (*n* = 132). When patients who developed POI were compared with patients who did not develop POI, modified CCI and hypertension suggested comparable modified CCI and hypertension prevalence between groups (see Appendix).

### Module D—GI Medical History

**Figure 4:**
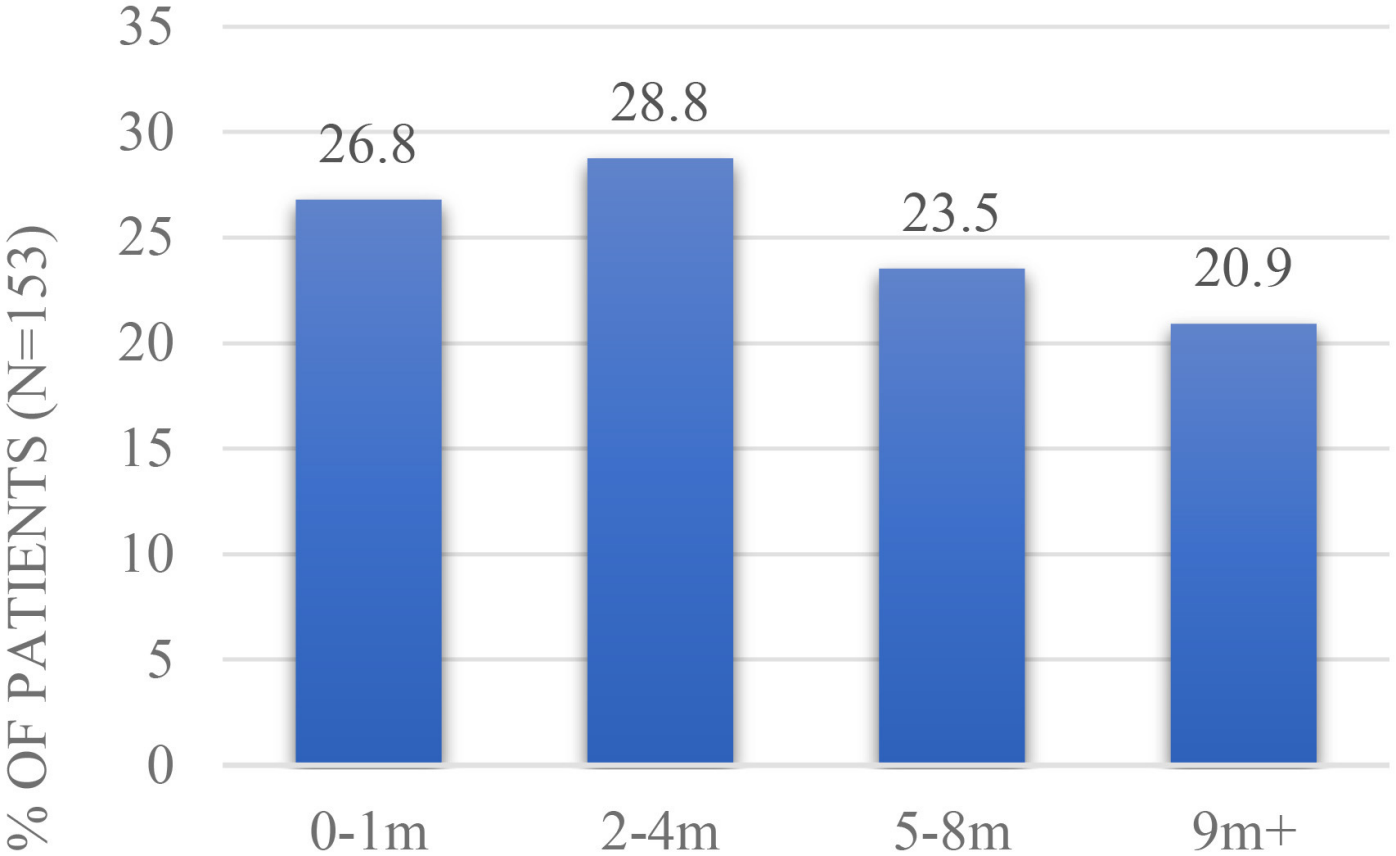
Duration of symptoms prior to diagnosis.

The median duration of symptoms prior to diagnosis was 3 months (IQR 1–8 months; range 0–96 months) (*n* = 153), see Figure 4 below. Only 5,8% of the patients (*n* = 9) had had a previous GI diagnosis namely irritable bowel syndrome (IBS): *n* = 3, inflammatory bowel disease (IBD): *n* = 5 and unknown: *n* = 1. 61,4% of the patients had had previous abdominal surgery (*n* = 145).

When patients who developed POI were compared with patients who did not develop POI, GI medical history suggested comparable median duration of symptoms prior to diagnosis, and comparable previous abdominal surgery (Appendix). Previous GI diagnosis was not analysed due to the low percentage (5,8%; *n* = 9) of patients that had had previous GI diagnosis.

### Module E—Pharmacotherapy

**Figure 5:**
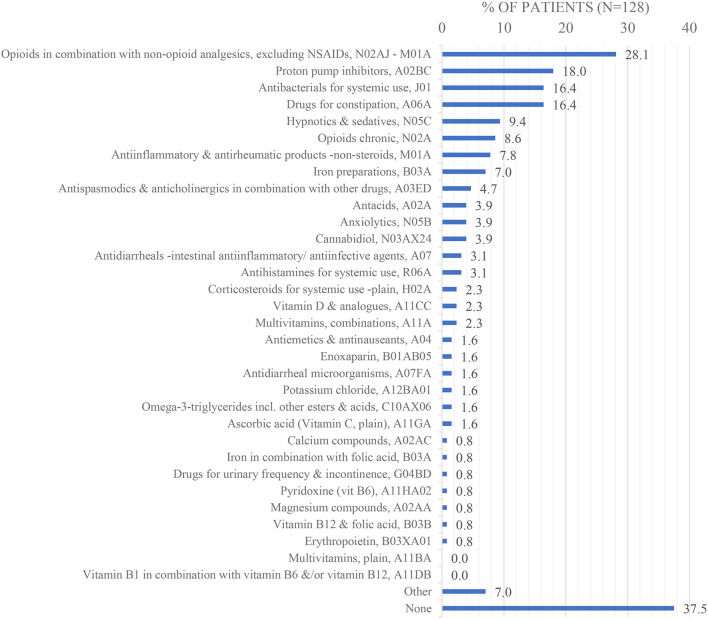
Acute preoperative pharmacotherapy.

Acute preoperative pharmacotherapy within 180 days before the surgery (*n* = 128; 17,4% missing data): as seen in Figure 5 the most commonly used medications were opioids in combination with non-opioid analgesics, excluding NSAIDs, N02AJ–M01A (28,1%).

**Figure 6:**
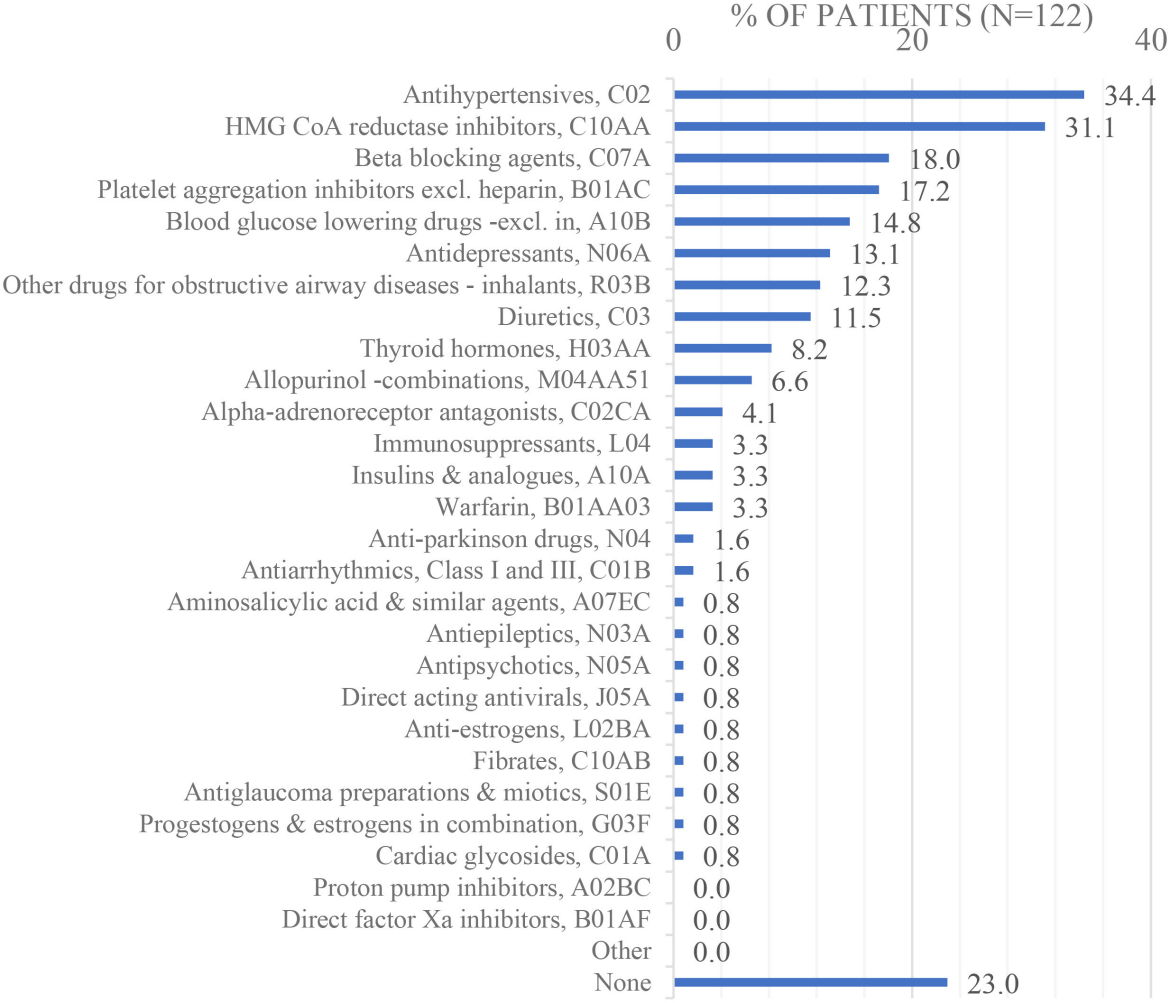
Chronic preoperative pharmacotherapy.

Chronic preoperative pharmacotherapy (*n* = 122; 21,3% missing data): as seen in Figure 6, the most commonly used medications were antihypertensives, C02 (34,4%), and 3-Hydroxy-3-Methyl-Glutaryl- Coenzyme A (HMG CoA) reductase inhibitors, C10AA (31,1%).

Neoadjuvant chemotherapy: 20,6% (*n* = 32) of patients received neoadjuvant chemotherapy and 79,35% of patients received adjuvant (*n* = 28) or no chemotherapy (*n* = 95) (see Appendix). Fifty six percent of patients receiving neoadjuvant chemotherapy had rectal cancer, 31% had left-sided colon cancer and 12% right-sided colon cancer. Sixty eight percent (*n* = 31; 3% missing data) of patients were treated with neoadjuvant 5-Fluorouracil (5-FU) in combination with folinic acid and oxaliplatin, L01BC02 + B03BB01 + L01XA03 (FOLFOX). Fifty six percent of patients received monoclonal antibodies concomitantly with chemotherapy (*n* = 18), of which *n* = 12 received bevacizumab (Avastan), L01XC07 and *n* = 6 received etuximab (Erbitux), L01XC06. Thirty seven percent of patients (*n* = 12) did not receive monoclonal antibodies (6% missing data). Fifty nine percent (*n* = 16 out of *n* = 27; 16% missing data) of patients had 1–6 chemotherapy cycles. Fourteen percent (*n* = 4 out of *n* = 28; 13% missing data) of patients had complications. Eighty five percent (*n* = 22 out of *n* = 26; 19% missing data) of patients completed the course of chemotherapy.

When patients who developed POI were compared with patients who did not develop POI, pharmacotherapy suggested comparable preoperative acute medication including opioids in combination with non-opioid analgesics, excluding NSAIDs, N02AJ–M01A; proton pump inhibitors, A02BC; antibacterials for systemic use, J01 and drugs for constipation, A06A (Appendix). Preoperative chronic medication including antihypertensives, C02; HMG CoA reductase inhibitors, C10AA; beta-blocking agents, C07A; platelet aggregation inhibitors excluding heparin, B01AC; blood glucose-lowering drugs—excluding insulin, A10B; antidepressants, N06A; and other drugs for obstructive airway diseases (inhalants), R03B, were also comparable (Appendix). Neoadjuvant chemotherapy suggested comparable chemotherapy in terms of location of malignancy, antineoplastic agents with *n* > 15, and chemotherapy course completed (Appendix). The following variables were not analysed due to either high levels of missing data or categories with *n* < 15 at the overall level: acute/chronic pharmacotherapy with *n* < 15; antineoplastic agents with *n* < 15; the number of chemotherapy cycles; monoclonal antibodies used concomitantly with chemotherapy; chemotherapy complications (and type; outcomes).

### Module F—Neoadjuvant Radiation Therapy and—Chemoradiation

30,3% (*n* = 47) of patients received neoadjuvant radiation therapy and 69,7% of patients (*n* = 108) received no radiation therapy. Eighty nine percent (*n* = 42/47) of patients had rectal malignancy. Seventy two percent (*n* = 34/47; 11% missing data) of patients received a concomitant sensitising antineoplastic agent. *n* = 38/47 of patients received neoadjuvant radiation therapy within 180 days before the surgery.

When patients who developed POI were compared with patients who did not develop POI, neoadjuvant radiation therapy suggested comparable radiation therapy (any and within 180 days; intent (long course); completion status and radiation therapy sensitising agent used (Appendix). The following variables were not analysed due to high levels of missing data or *n* < 15 at the overall level: radiation therapy sensitising agent type; radiation therapy complications (and type; outcomes).

The Appendix shows the univariate analysis of preoperative risk factors for POI and the multivariable analysis with ≤ 5 qualifying parameters.

### Multivariable Analysis

There was no significant association between POI and the following variables but *p* < 0,20 so these variables were considered for multivariable analysis together with gender (men) and previous smoking status (Appendix):

- Ethnicity (*p* = 0,17)
- Chronic drugs: HMG CoA reductase inhibitors, C10AA (*p* = 0,19); blood glucose-lowering drugs, excluding insulin, A10B (*p* = 0,15); and antidepressants, N06A (*p* = 0,18)
- Radiation therapy: within 180 days before surgery (*p* = 0,091); intent (long course) (*p* = 0,088); and completion status (*p* = 0,13).

Radiation intent, therapy (yes or no), and completion were confounded so only whether radiation was received or not was included in a multivariate model (34). The seven remaining variables and nine parameters were too many to be estimated given the size of the smallest outcome group (*n* = 56). Sample size considerations allowed the estimation of at most five parameters, so the variables with the least significant *p*-values were excluded. A model with ≤ 5 parameters showed non-significance for all variables at the 5% significance level. Lastly, the most non-significant variables were removed one at a time until only previous smoking status remained.

## Discussion

### Colorectal Cancer Presentation

In the present study, most patients diagnosed with CRC (34,2%) were between 60 and 69 years of age and 52,3% of the patients were men. The majority of the patients were white (69,7%). This disease presentation is comparable to international reports and to the patient presentation reported by a retrospective review of a private healthcare funder's database in SA (2). CRC is more common in the white population of SA than the black African population and is associated with a Western diet and lifestyle (38).

### POI Incidence

In the present study, the POI incidence rate seen in the study population was 36% (56 POI out of 155 cases) which was higher than anticipated (refer to Table 1). The incidence rate previously reported at the same institution was 13,7% (19 POI out of 139 elective colorectal resections between December 2016 to November 2017) (20). The POI incidence rate reported in a literature review of 11 studies ranged between 2 and 54%, with the median incidence rate reported as 10,3% after colorectal surgery (5). The wide range of incidence rates reported by different studies/centres may be attributed to the lack of standardised definitions for POI and differences in clinical settings which leads to misclassification bias which is a threat to external validity because it hinders the head-to-head comparison between studies (14, 16, 18). International consensus on which definitions to use was recently reached and will make it easier to discover the true incidence of POI going forward (19).

### Preoperative Risk

Previous studies found many preoperative variables to be associated with the development of POI after abdominal surgery (4, 10, 11, 13, 15–18, 35–37), as can be seen in Table 2.

In the present study, univariate analysis of the preoperative modules identified only being a man and previous smoking status as risk factors for POI. Men were found to be in themselves an independent risk factor by several other studies (11, 15–18). Smoking history was also previously found to be an independent risk factor by a large multicentre study (35), but there exists a shortage of information on why previous smokers may be predisposed to develop POI. Although the majority of the patients were current consumers of alcohol there was no significant association between POI and current alcohol use. In the present study, none of the results of a multivariate model with ≤ 5 qualifying parameters showed significance at a 5% significance level.

The association between men and POI may be attributed to an increased inflammatory response to surgery which is described in the literature (39). Besides the local damage caused during surgical resection, intestinal handling, and manipulation during surgery, even gentle inspection of the intestine at the very beginning of the surgical procedure also triggers the body's innate immune system within hours of abdominal surgery (14). The innate immune system is a non-specific cascade of events instrumental in the host defence system and in initiating the inflammatory response and intestinal inflammation after abdominal surgery that occurs throughout the entire gastrointestinal tract (GIT) and is not only limited to the resected segment (14). The main players of the innate immune system are peritoneal mast cells and resident dormant macrophages (40). Mast cell activation triggers a short period of increased mucosal permeability allowing access to intraluminal bacteria or bacterial products to pass through which will, in turn, activate resident macrophages in the muscularis externa (14). The subsequent release of pro-inflammatory cytokines and chemokines (arachidonic acid pathway activation) causes the up-regulation of intracellular adhesion molecules in the endothelium (7, 14). Phagocytes present throughout the gut are triggered, resulting in a migration of leucocytes to the muscularis externa (13). The release of nitric oxide, cyclooxygenase-2 (COX-2) metabolites, and prostaglandins by these phagocytes prevents peristalsis by inhibiting smooth muscle contractility directly which leads to POI (14, 39, 41).

It is noteworthy that in the present study the majority of patients (60,0%) had an open surgery technique but no significant association was found between POI and surgical access. This is in contrast with four studies that all found an open technique to be an independent risk factor for POI (11, 16, 18, 35) and one study that found open surgery to be associated with POI (15) (see Table 2).

Previous studies found chronic preoperative narcotic use (13) and neoadjuvant chemotherapy (18) to be independent risk factors for POI. In the present study, 28,1% of patients received opioids in combination with non-opioid analgesics, excluding NSAIDs, N02AJ—M01A (*n* = 36), which was not found to be a significant risk factor in univariate analysis. Only 8,6% of patients (*n* = 11) received chronic preoperative opioids, N02A. Neither neoadjuvant chemotherapy nor chemoradiation was found to be a significant risk factor for POI.

### Preventative Strategies and Treatment of POI

There is evidence in the literature that overall compliance of more than 70% to the ERAS programme as well as minimally invasive surgery helped prevent POI and therefore significantly minimised POI-associated medical complications (19, 41). Treatment should include components of ERAS aimed at preventing the inflammatory response early on in the inflammatory cascade activated by intestinal handling during surgery (14, 19). Modulation of vagal afferents with chewing gum (42) and coffee (43) can be considered as a method of attenuating this inflammatory response, since the release of Acetylcholine (ACh) can reduce cytokine release by intestinal macrophages (7, 19). The use of Non-steroidal antiinflammatory drugs (NSAIDs), also as an opioid-restrictive measure (19) may be most effective early in the inflammatory cascade to prevent POI but may even become harmful when inflammation is already well-established and an assortment of inflammatory mediators are released (14). NSAIDs fall under immune-modulating therapy, which, if used too late in the inflammatory cascade, may increase the risk of anastomotic leak.

The high incidence of POI at the study hospital was identified in the previous study as one of the reasons for prolonged LOS at the institution and some postoperative components of ERAS including limitation of fluid and opioid-sparing strategies were implemented at the study hospital (20). Due to challenges like high cost and a shortage in resources in SA (44), full implementation of all ERAS components has not been possible yet (20, 44).

## Study Strengths and Limitations

### Study Design and Available Variables

All the relevant perioperative variables that could have been investigated based on known risk factors for POI were not recorded on the original SAMRC CRC study REDCap database because the cohort was constructed before the conception of this study and data were collected for another purpose (21). This is a common strength but also a disadvantage of a retrospective cohort study (45). The cohort study design itself limited research selection bias because the outcome of interest (POI) was not the original reason for the SAMRC CRC cohort to be constructed or the preoperative assessment forms to be completed and the patients themselves were selecting for POI and no POI. The study design may have led to information and confusion bias. The use of a standardised preoperative assessment form minimised information bias. A standardised definition was utilised by the Colorectal Unit to classify patients with the outcome of POI or no POI. The design of the data collection instrument was based on the study protocol to ensure the reliability of data and consistency of data collection to minimise implementation bias. Researcher bias was also minimised due to the study design. This threat to internal (historical) validity was partly overcome by supplementing cohort data from the SAMRC CRC study REDCap database with data from standardised Preoperative Assessment Clinic patient assessment forms. Even with this supplementation, there was still very limited data available on the preoperative and surgical management of patients. Due to *n* < 15 per type of surgical procedure. 18,7% of data were not included in the analyses. Specific procedure types were previously associated with the postoperative development of ileus by other studies (11, 15, 36). We did not attempt to completely eliminate confusion bias because the aim of the study was focused on preoperative risk factors associated with the development of the outcome POI.

### Confounding Variables

It is difficult in a retrospective cohort study to control for all confounding variables. There is a lack of randomisation following a cohort study design which may also lead to heterogeneity—the patients themselves are selecting POI or no POI. This allows confounding bias to influence the results and may also compromise the internal validity of data (45). The chi-squared test was utilised to examine each pair of variables for possible confounding or to test for “goodness of fit” before combining qualifying study variables into a multivariable model.

### Sample Size and Statistical Analysis

The SAMRC CRC longitudinal study was conducted over a specified time period, limiting the sample size of this and any further cohort studies. The decision to add the postoperative complications anastomotic leak and non-therapeutic surgery to the exclusion criteria were based on valid reasons, but significantly reduced the number of qualifying surgeries. Anastomotic leak is considered to be a secondary cause of POI (19, 46). Any surgery with less intense nociceptive stimulation usually only causes the activation of spinal afferents (14), which is only one of many identified neurohormonal pathways leading to inhibition of GI motility (14, 47, 48). A pre-therapeutic or secondary surgery is, therefore, less likely to lead to POI as a complication. The inclusion of patients with stoma formation in the current study (63,9% of patients) was necessary to preserve an adequate sample size. Although stoma formation was found to be associated with the development of POI by other studies (15, 17), there was no significant association between POI and stoma formation in the current study. The inclusion of such a high percentage of patients with stoma formation into this study, may however still be problematic due to stoma-related obstruction. POI was clinically diagnosed according to the ACS-NSQIP definition: “any NGT use or NPO status on postoperative day 4 or later” (18) with the radiological exclusion of small bowel obstruction which is a strength of this study and avoided this potential misclassification. The exclusion of *n* < 15 from the statistical analysis is a strength of the study because no reliable inference could be made based on such small groups. The sample size of the current study allowed the estimation of Relative Risks of 1,45 or greater with 80% power at the 5% significance level. This limits the RR of variables that could have been detected and associations, which could have led to recommendations for action may have been missed as a result. Lastly, by using an external statistician to accurately define the statistical significance of the data obtained, confirmation bias where data with borderline statistical significance is discussed was avoided.

### Study Population

The study is only representative of a single private healthcare institution in SA. Due to vast socio-economic and ethnic differences between the private and public sector in SA ecological validity is compromised. Although multi-centre data and data from the public sector would be valuable—comparing across institutions may have also introduced additional levels of bias. In particular collecting data from a single-centre institution minimised misclassification bias in the current study.

## Recommendations

Intra- and postoperative variables also play a role in the development of POI and there will be merit in also investigating these variables in further studies in SA to determine, which were associated with POI as complication post elective therapeutic CRC resection. The high incidence rate of POI in the present study's patient population however shows that implementation of an ERAS programme, or at least components thereof, should happen regardless. There is substantial evidence in the literature of the many clinical benefits and cost savings after successful implementation of ERAS and the establishment of an ERAS centre at the study hospital would also entail the standardisation of perioperative care and continuation of a formal prospective data collection process which will reduce limitations encountered during this study and further research on POI. It is strongly recommended that future research also be conducted on POI in the public healthcare sector in SA because this is where the majority of the population access their healthcare. The multidisciplinary, longitudinal cohort study on CRC in Johannesburg that formed the cohort for the current study also used selected public sector study sites for recruitment. A future retrospective study to determine the POI incidence rate for these public sector hospitals would be valuable.

## Conclusion

More studies are needed to adequately determine local perioperative risk factors for POI, however, the high local incidence rate of POI in this patient population shows that intervention is required to improve postoperative outcomes. This study suggests that men and all patients with a history of smoking undergoing CRC resection are at higher risk to develop POI. Preoperative recommendations for these patients with the intention to prevent POI should include instructions initiating the activation of therapeutic pathways like the ERAS programme.

## Data Availability Statement

The datasets presented in this study can be found in online repositories. The names of the repository/repositories and accession number(s) can be found in the article/Supplementary Material.

## Ethics Statement

The studies involving human participants were reviewed and approved by University of the Witwatersrand Johannesburg, Human Research Ethics Committee (Medical), clearance certificate no. M190526 and Sefako Makgatho Health Sciences University Research Ethics Committee, reference no. SMUREC/P/55/2019: PG. The SA-MRC Colorectal Cancer database that formed the study population was prospective and all participants did sign an informed consent form at the time of recruitment to the study (University of the Witwatersrand Johannesburg, Human Research Ethics Committee (Medical), clearance certificate no. M150446). Since this study was retrospective, there was no additional consent form. The ethics committee waived the requirement of written informed consent for participation.

## Author Contributions

EW was the principal researcher and lead author of the manuscript. NS and VA supervised the study. NS, VA, and BB participated in the writing and editing of the paper. All authors listed have made a substantial, direct and intellectual contribution to the work, and approved it for publication.

## Conflict of Interest

The authors declare that the research was conducted in the absence of any commercial or financial relationships that could be construed as a potential conflict of interest.
