# Supplementary material for: Men and Those With a History of Smoking Are Associated With the Development of Postoperative Ileus Following Elective Colorectal Cancer Resection at a Private Academic Hospital in Johannesburg, South Africa: A Retrospective Cohort Study
Source: Front Surg. 2021 Jun 15;8:667124. doi: 10.3389/fsurg.2021.667124 (PMC8239403; doi:10.3389/fsurg.2021.667124)
Supplement: Supplementary file 1 [file Table_1.docx]

**Appendix: Supplementary Data**

Table 1: Cohort study group description and univariate and multivariable analysis of preoperative risk factors for POI

| **Variable** | **Category** | **Overall**  **n=155** | | **No POI**  **n=99** | | **POI**  **n=56** | | **% POI**  **n=36,1** | **Univariate analysis** | | | | **Multivariable analysis model with ≤ 5 qualifying parameters** | | | |
| --- | --- | --- | --- | --- | --- | --- | --- | --- | --- | --- | --- | --- | --- | --- | --- | --- |
|  |  | **n** | **%** | **n** | **%** | **n** | **%** |  | **p-value** | **RR for POI** | **95% CI for RR** | | **p-value** | **RR for POI** | **95% CI for RR** | |
| **Module A – Patient Demographics** | | | | | | | | | | | | | | | | |
| Age at time of surgery | 18–49y  50–59y  60–69y  70y+ | 32  40  53  30 | 20,6  25,8  34,2  19,4 | 22  25  36  16 | 22  25  36  16 | 10  15  17  14 | 18  27  30  25 | 31,3  37,5  32,1  46,7 | 0,58  0,94  0,22 | 1  1,20  1,03  1,49 | Reference | |  |  |  |  |
|  |  |  |  |  |  |  |  |  |  |  | 0,63  0,54  0,79 | 2,30  1,96  2,83 |  |  |  |  |
| Gender | Male  Female | 81  74 | 52,3  47,7 | 44  55 | 44  56 | 37  19 | 66  34 | 45,7  25,7 | **0,013** | 1  **0,56** | Reference | | 0,12 | 1  0,68 | Reference | |
|  |  |  |  |  |  |  |  |  |  |  | **0,36** | **0,89** |  |  | 0,41 | 1,11 |
| Ethnicity | White  Black  Indian  Other | 108  20  19  8 | 69,7  12,9  12,3  5,2 | 65  13  15  6 | 66  13  15  6 | 43  7  4  2 | 77  13  7  4 | 39,8  35,0  21,1 | 0,69  **0,17** | 1  0,88  0,53 | Reference | |  |  |  |  |
|  |  |  |  |  |  |  |  |  |  |  | 0,46  0,21 | 1,67  1,30 |  |  |  |  |
| Tumour stage | Local  Regional  Distant | 65  61  29 | 41,9  39,4  18,7 | 40  41  18 | 40  41  18 | 25  20  11 | 45  36  20 | 38,5  32,8  37,9 | 0,51  0,96 | 1  0,85  0,99 | Reference | |  |  |  |  |
|  |  |  |  |  |  |  |  |  |  |  | 0,53  0,56 | 1,37  1,72 |  |  |  |  |
| Location of malignancy: | Colon (right) No  Colon (right) Yes  Colon (left) No  Colon (left) Yes  Colon (rectum) No  Colon (rectum) Yes  Synchronous lesion No  Synchronous lesion Yes | 131  24  113  42  65  90  154  1 | 84,5  15,5  72,9  27,1  41,9  58,1  99,4  0,6 | 84  15  69  30  44  55  98  1 | 85  15  70  30  44  56  99  1 | 47  9  44  12  21  35  56  0 | 84  16  79  21  38  63  100  0 | 35,9  37,5  38,9  28,6  32,3  38,9 | 0,88  0,25  0,41 | 1  1,05  1  0,73  1  1,20 | Reference | |  |  |  |  |
|  |  |  |  |  |  |  |  |  |  |  | 0,59 | 1,84 |  |  |  |  |
|  |  |  |  |  |  |  |  |  |  |  | Reference | |  |  |  |  |
|  |  |  |  |  |  |  |  |  |  |  | 0,43 | 1,25 |  |  |  |  |
|  |  |  |  |  |  |  |  |  |  |  | Reference | |  |  |  |  |
|  |  |  |  |  |  |  |  |  |  |  | 0,78 | 1,86 |  |  |  |  |
| Surgical procedure | Low anterior resection (LAR) (rectal)  Right hemicolectomy (right sided)  Abdominal perineal resection (APR) (rectal)  Other (n<15 per type of surgical procedure) | 81  22  23  33 | 52,3  14,2  14,8  18,7 | 50  14  16 | 51  14  16 | 31  8  7 | 55  14  13 | 38,3  36,4  30,4 | 0,56  0,98  0,55 | 1,13  1,01  0,82 | 0,74  0,55  0,43 | 1,73  1,83  1,58 |  |  |  |  |
| Surgical access | Open operation  Laparoscopic completed  Laparoscopic assisted open operation  Laparoscopic converted to open | 93  57  3  2 | 60  36,8  1,9  1,3 | 63  33  2  1 | 64  33  2  1 | 30  24  1  1 | 54  43  2  2 | 32,3  42,1 | 0,22 | 1  1,31 | Reference | |  |  |  |  |
|  |  |  |  |  |  |  |  |  |  |  | 0,85 | 1,99 |  |  |  |  |
| Stoma Formation | No  Yes | 56  99 | 36,1  63,9 | 37  62 | 37  63 | 19  37 | 34  66 | 33,9  37,4 | 0,67 | 1  1,10 | Reference | |  |  |  |  |
|  |  |  |  |  |  |  |  |  |  |  | 0,71 | 1,72 |  |  |  |  |
| Cancer-related complication/s at surgery | No  Yes | 123  32 | 79,4  20,6 | 79  20 | 80  20 | 44  12 | 79  21 | 35,8  37,5 | 0,86 | 1  1,05 | Reference | |  |  |  |  |
|  |  |  |  |  |  |  |  |  |  |  | 0,63 | 1,74 |  |  |  |  |
| **Module B – Functional Status** | | | | | | | | | | | | | | | | |
| Body Mass Index (BMI) within 42 days before the surgery | < 25  ≥ 25  Missing | 46  83  26 | 29,7  53,5  16,8 | 31  48 | 39  61 | 15  35 | 30  70 | 32,6  42,2 | 0,30 | 1 | Reference | |  |  |  |  |
|  |  |  |  |  |  |  |  |  |  | 1,29 | 0,80 | 2,10 |  |  |  |  |
| Smoking status | Never  Current  Previous | 84  20  51 | 54,2  12,9  32,9 | 59  16  24 | 60  16  24 | 25  4  27 | 45  7  48 | 29,8  20,0  52,9 | 0,41  **0,0069** | 1  0,67  1,78 | Reference | | 0,19  0,33 | 1  0,53  1,28 | Reference | |
|  |  |  |  |  |  |  |  |  |  |  | 0,26  1,17 | 1,71  2,70 |  |  | 0,21  0,78 | 1,37  2,10 |
| Alcohol consumption | Never  Current  Previous | 44  98  13 | 28,4  63,2  8,4 | 28  61  10 | 28  62  10 | 16  37  3 | 29  66  5 | 36,4  37,8  23,1 | 0,87  0,40 | 1  1,04  0,63 | Reference | |  |  |  |  |
|  |  |  |  |  |  |  |  |  |  |  | 0,65  0,22 | 1,66  1,84 |  |  |  |  |
| ASA grading within 42 days before the surgery | Grade I, patient is a completely healthy fit patient.  Grade II, patient has mild systemic disease.  Grade III, patient has severe systemic disease, not incapacitating  Grade IV, patient has incapacitating disease, a constant threat to life  Grade V, a moribund patient who is not expected to live 24 hours with or without the surgery  Missing | 44  43  4  0  0  64 | 28,4  27,7  2,6  0  0  41,3 |  |  |  |  |  |  |  |  |  |  |  |  |  |
| NYHA classification within 42 days before the surgery | I, no symptoms and no limitation in ordinary physical activity, e.g., shortness of breath when walking, climbing stairs etc.  II, mild symptoms and light limitation during ordinary activity  III, marked limitation in activity due to symptoms, even during less-than-ordinary activity, e.g., walking short distances  IV, severe limitations. Experience symptoms even at rest  Missing | 64  33  0  0  58 | 41,3  21,3  0  0  37,4 |  |  |  |  |  |  |  |  |  |  |  |  |  |
| **Module C – Modified Charlson comorbidity index (modified CCI); Hypertension** | | | | | | | | | | | | | | | | |
| Modified Charlson Comorbidity Index (CCI) | 0  1  2 or more  Missing | 93  26  13  23 | 60,0  16,8  8,4  14,8 | 56  13  11  19 | 70  16  14 | 37  13  2  4 | 71  25  4 | 39,8  38,5 | 0,89 | 1  0,97 | Reference | |  |  |  |  |
|  |  |  |  |  |  |  |  |  |  |  | 0,60 | 1,55 |  |  |  |  |
| Hypertension | No  Yes  Missing | 83  49  23 | 53,5  31,6  14,8 | 50  30  19 | 63  38 | 33  19  4 | 63  37 | 39,8  38,8 | 0,91 | 1  0,98 | Reference | |  |  |  |  |
|  |  |  |  |  |  |  |  |  |  |  | 0,63 | 1,51 |  |  |  |  |
| **Module D – Gastrointestinal medical history** | | | | | | | | | | | | | | | | |
| Duration of symptoms prior to diagnosis | 0-1 Month  2-4 Month  5-8 Month  9 Months plus  Missing | 41  44  36  32  2 | 26,8  28,8  23,5  20,9  1,3 | 27  23  25  23  1 | 28  23  26  23 | 14  21  11  9  1 | 25  38  20  16 | 34,1  47,7  30,6  28,1 | 0,21  0,74  0,59 | 1  1,40  0,89  0,82 | Reference | |  |  |  |  |
|  |  |  |  |  |  |  |  |  |  |  | 0,83  0,47  0,41 | 2,36  1,72  1,66 |  |  |  |  |
| Previous gastrointestinal diagnosis | None  Irritable bowel syndrome (IBS)  Inflammatory bowel disease (IBD)  Any (IBS + IBD)  Unknown | 146  3  5  9  1 | 94,2  1,7  3,4  5,8  0,6 | 92  7 | 93  7 | 54  2 | 96  4 |  |  |  |  |  |  |  |  |  |
| Previous abdominal surgery | No  Yes  Missing | 56  89  10 | 36,1  57,4  6,5 | 36  55  8 | 40  60 | 20  34  2 | 37  63 | 35,7  38,2 | 0,76 | 1  1,07 | Reference | |  |  |  |  |
|  |  |  |  |  |  |  |  |  |  |  | 0,69 | 1,66 |  |  |  |  |
| **Module E – Pharmacotherapy** | | | | | | | | | | | | | | | | |
| Acute preoperative pharmacotherapy within 180 days before the surgery | Opioids & non-opioid analgesics, N02AJ - M01A  Proton pump inhibitors, A02BC  Antibacterials for systemic use, J01  Drugs for constipation, A06A  Hypnotics and sedatives, N05C  Opioids chronic, N02A  Antiinflammatory and antirheumatic products -non-steroids, M01A  Iron preparations, B03A  Antispasmodics and anticholinergics in combination with Other drugs, A03ED  Antacids, A02A  Anxiolytics, N05B  Cannabidiol, N03AX24  Antidiarrheals -intestinal antiinflammatory/ antiinfective Agents, A07  Antihistamines for systemic use, R06A  Corticosteroids for systemic use -plain, H02A  Vitamin D and analogues, A11CC  Multivitamins, combinations, A11A  Antiemetics and antinauseants, A04  Enoxaparin, B01AB05  Antidiarrheal microorganisms, A07FA  Potassium chloride, A12BA01  Omega-3-triglycerides incl. other esters and acids, C10AX06  Ascorbic acid (Vitamin C, plain), A11GA  Calcium compounds, A02AC  Iron in combination with folic acid, B03A  Drugs for urinary frequency and incontinence, G04BD  Pyridoxine (vit B6), A11HA02  Magnesium compounds, A02AA  Vitamin B12 and folic acid, B03B  Erythropoietin, B03XA01  Multivitamins, plain, A11BA  vitamin B1 in combination with vitamin B6 and/or vitamin B12, A11DB  other  none  Unknown | 36  23  21  21  12  11  10  9  6  5  5  5  4  4  3  3  3  2  2  2  2  2  2  1  1  1  1  1  1  1  0  0  9  48  27 | 28,1  18,0  16,4  16,4  9,4  8,6  7,8  7,0  4,7  3,9  3,9  3,9  3,1  3,1  2,3  2,3  2,3  1,6  1,6  1,6  1,6  1,6  1,6  0,8  0,8  0,8  0,8  0,8  0,8  0,8  0,0  0,0  7,0  37,5 | 23  13  10  15  8  6  7  6  4  4  3  4  3  2  3  1  2  2  2  2  2  1  1  1  0  0  0  0  1  1  0  0  5  28  20 | 29,1  16,5  12,7  19,0  10,1  7,6  8,9  7,6  5,1  5,1  3,8  5,1  3,8  2,5  3,8  1,3  2,5  2,5  2,5  2,5  2,5  1,3  1,3  1,3  0,0  0,0  0,0  0,0  1,3  1,3  0,0  0,0  6,3  35,4 | 13  10  11  6  4  5  3  3  2  1  2  1  1  2  0  2  1  0  0  0  0  1  1  0  1  1  1  1  0  0  0  0  4  20  7 | 26,5  20,4  22,4  12,2  8,2  10,2  6,1  6,1  4,1  2,0  4,1  2,0  2,0  4,1  0,0  4,1  2,0  0,0  0,0  0,0  0,0  2,0  2,0  0,0  2,0  2,0  2,0  2,0  0,0  0,0  0,0  0,0  8,2  40,8 | 36,1 | >0,99 | 1,00 | 0,61 | 1,64 |  |  |  |  |
| Chronic preoperative pharmacotherapy (refer to definition) | Antihypertensives, C02  HMG CoA reductase inhibitors, C10AA  Beta blocking agents, C07A  Platelet aggregation inhibitors excl. heparin, B01AC  Blood glucose lowering drugs -excl. in, A10B  No  Yes  Antidepressants, N06A  Other drugs for obstructive airway diseases – inhalants, R03B  Diuretics, C03  Thyroid hormones, H03AA  Allopurinol -combinations, M04AA51  Alpha-adrenoreceptor antagonists, C02CA  Immunosuppressants, L04  Insulins and analogues, A10A  Warfarin, B01AA03  Anti-parkinson drugs, N04 | 42  38  22  21  18  16  15  14  10  8  5  4  4  4  2 | 34,4  31,1  18,0  17,2  14,8  13,1  12,3  11,5  8,2  6,6  4,1  3,3  3,3  3,3  1,6 | 26  21  14  14  9  8  12  9  6  3  4  4  2  3  2 | 35,6  28,8  19,2  19,2  12,3  11,0  16,4  12,3  8,2  4,1  5,5  5,5  2,7  4,1  2,7 | 16  17  8  7  9  8  3  5  4  5  1  0  2  1  0 | 32,7  34,7  16,3  14,3  18,4  16,3  6,1  10,2  8,2  10,2  2,0  0,0  4,1  2,0  0,0 | 38,1  44,7  36,4  33,3  50,0  50,0  20,0 | 0,75  **0,19**  0,98  0,78  **0,15**  **0,18**  0,23 | 1,08  1,34  1,01  0,91  1,46  1,45  0,53 | 0,68  0,87  0,55  0,48  0,87  0,84  0,19 | 1,70  2,08  1,83  1,74  2,44  2,49  1,49 | 0,18 | 1  1,37 | Reference | |
|  |  |  |  |  |  |  |  |  |  |  |  |  |  |  | 0,86 | 2,17 |
|  | Antiarrhythmics, Class I and III, C01B  Aminosalicylic acid and similar agents, A07EC  Antiepileptics, N03A  Antipsychotics, N05A  Direct acting antivirals, J05A  Anti-estrogens, L02BA  Fibrates, C10AB  Antiglaucoma preparations and miotics, S01E  Progestogens and estrogens in combination, G03F  Cardiac glycosides, C01A  Proton pump inhibitors, A02BC  Direct factor Xa inhibitors, B01AF  Other  None  Unknown | 2  1  1  1  1  1  1  1  1  1  0  0  0  28  33 | 1,6  0,8  0,8  0,8  0,8  0,8  0,8  0,8  0,8  0,8  0,0  0,0  0,0  23,0 | 1  1  0  0  0  1  0  1  1  1  0  0  0  16  26 | 1,4  1,4  0,0  0,0  0,0  1,4  0,0  1,4  1,4  1,4  0,0  0,0  0,0  21,9  35,6 | 1  0  1  1  1  0  1  0  0  0  0  0  0  12  7 | 2,0  0,0  2,0  2,0  2,0  0,0  2,0  0,0  0,0  0,0  0,0  0,0  0,0  24,5  14,3 |  |  |  |  |  |  |  |  |  |
| Neoadjuvant chemotherapy (for any time) | No  Adjuvant chemotherapy  No Chemotherapy  Yes | 123  28  95  32 | 79,35  20,64 | 77  22 | 78  22 | 46  10 | 82  18 | 37,4  31,3 | 0,72 | 1  0,90 | Reference | |  |  |  |  |
|  |  |  |  |  |  |  |  |  |  |  | 0,50 | 1,61 |  |  |  |  |
| Location of malignancy | Adjuvant- & no chemotherapy  Colon right  Colon left  Rectum | 123  4  10  18 | 79,35  2,58  6,45  11,62 | 77  4  8  10 | 78  4  8  10 | 46  0  2  8 | 82  0  4  14 | 37,4  44,4 | 0,41 | 1  1,27 | Reference | |  |  |  |  |
|  |  |  |  |  |  |  |  |  |  |  | 0,72 | 2,23 |  |  |  |  |
| Number of cycles | Adjuvant- & no chemotherapy  1–6  8–12  Missing | 123  16  11  5 | 79,35  10,32  7,10  3,23 | 77  10  10  2 | 79  10  10 | 46  6  1  3 | 87  11  2 |  |  |  |  |  |  |  |  |  |
| Antineoplastic agent, L01 | Adjuvant & no chemotherapy  5-FU + folinic acid + oxaliplatin (FOLFOX), L01BC02 + B03BB01 + L01XA03  Capecitabine + oxaliplatin (XELOX), L01BC06 + L01XA03  Capecitabine (Xeloda), L01BC06  Folinic acid + 5-FU and irinotecan (FOLFIRI), B03BB01 + L01BC02 + L01XX19  Capecitabine + irinotecan (XELIRI), L01BC06 + L01XX19  5 FU, L01BC02  5 FU + folinic acid (leucovorin), L01BC02 + B03BB01  Folinic acid + 5-FU + oxaliplatin and irinotecan (FOLFOXIRI), B03BB01 + L01BC02  TAS-102 + trifluridine/ tipiracil (Lonsurf), L01BC59  Irinotecan, L01XX19  Other  Unknown | 123  21  9  1  1  0  0  0  0  0  0  0  1 | 78,85  13,46  5,77  0,64  0,64  0,00  0,00  0,00  0,00  0,00  0,00  0,00  0,64 | 77  17  4  1  0  0  0  0  0  0  0  0  1 | 79  17  4  1  0  0  0  0  0  0  0  0 | 46  4  5  0  1  0  0  0  0  0  0  0 | 82  7  9  0  2  0  0  0  0  0  0  0 | 37,4  19,0 | 0,20 | 1  0,55 | Reference | |  |  |  |  |
|  |  |  |  |  |  |  |  |  |  |  | 0,22 | 1,38 |  |  |  |  |
| Complications and outcome | Adjuvant- & no chemotherapy  No  Yes  Missing | 123  24  4  4 | 79,35  15,48  2,58  2,58 | 77  16  4  2 | 79  16  4 | 46  8  0  2 | 85  15  0 |  |  |  |  |  |  |  |  |  |
| Monoclonal antibodies, L01XC | Adjuvant & no chemotherapy  Bevacizumab (Avastan), L01XC07  Cetuximab (Erbitux), L01XC06  No monoclonal antibodies  Missing | 123  12  6  12  2 | 79,35  7,74  3,87  7,74  1,29 | 77  8  4  8  2 | 79  8  4  8 | 46  4  2  4  0 | 82  7  4  7 |  |  |  |  |  |  |  |  |  |
| Chemotherapy course completed | Adjuvant & no chemotherapy  Yes  No  Missing | 123  22  4  6 | 79,35  14,19  2,58  3,87 | 77  15  4  3 | 80  16  4 | 46  7  0  3 | 87  13  0 | 37,4  31,8 | 0,80 | 1  0,92 | Reference | |  |  |  |  |
|  |  |  |  |  |  |  |  |  |  |  | 0,47 | 1,79 |  |  |  |  |
| **Module F – Neoadjuvant radiation and -chemoradiation** | | | | | | | | | | | | | | | | |
| Radiation therapy within the last 180 days before the surgery | No  Adjuvant radiation therapy  Radiation therapy not within 180 days  No radiation therapy | 117  0  9  108 | 75,48 | 70 | 71 | 47 | 84 | 40,2 |  | 1 | Reference | |  | 1 | Reference | |
|  |  |  |  |  |  |  |  |  |  |  |  |  |  |  |  |  |
|  | Yes | 38 | 24,52 | 29 | 29 | 9 | 16 | 23,7 | **0,091** | 0,59 | 0,32 | 1,09 | **0.20** | 0,66 | 0,36 | 1,24 |
| Location of malignancy (for any time) | No radiation therapy  Colon right  Colon left  Rectum | 108  0  5  42 | 69,68  0,00  3,23  27,10 | 65  0  3  31 | 66  0  3  31 | 43  0  2  11 | 77  0  4  20 |  |  |  |  |  |  |  |  |  |
| Intent of radiation within the last 180 days before the surgery | Radiation therapy not with 180 days & no radiation  Long course  Short course  Palliative | 117  35  2  1 | 75,48  22,58  1,29  0,65 | 70  27  1  1 | 71  27  1  1 | 47  8  1  0 | 84  14  2  0 | 40,2  22,9 | **0,088** | 1  0,57 | Reference | |  |  |  |  |
|  |  |  |  |  |  |  |  |  |  |  | 0,30 | 1,09 |  |  |  |  |
| Radio-sensitising chemotherapeutic agent used (for any time) | No radiation therapy  No  Yes  Missing | 108  4  34  9 | 69,68  2,58  21,94  5,81 | 65  7  24  3 | 68  7  25 | 43  2  10  1 | 78  4  18 | 39,8  29,4 | 0,30 | 1  0,74 | Reference | |  |  |  |  |
|  |  |  |  |  |  |  |  |  |  |  | 0,42 | 1,31 |  |  |  |  |
| Type of radio-sensitising chemotherapeutic agent used (for any time) | No radiation therapy  No sensitising agent  Capecitabine (oral) (Xeloda), L01BC06  5 FU + folinic acid (leucovorin) (IV), L01BC02 + B03BB01  5-FU + folinic acid + oxaliplatin (FOLFOX), L01BC02 + B03BB01 + L01XA03  Folinic acid + 5-FU and irinotecan (FOLFIRI), B03BB01 + L01BC02 + L01XX19  Oxaliplatin, L01XA03  Unknown | 108  4  18  10  1  1  0  4 | 69,68  2,58  11,61  6,45  0,65  0,65  0,0 | 65  7  13  8  0  1  0  2 | 67  7  13  8  0  1  0 | 43  2  5  2  1  0  0  2 | 80  4  9  4  2  0  0 |  |  |  |  |  |  |  |  |  |
| Complications within the last 180 days before the surgery | Radiation therapy not with 180 days & no radiation  No  Yes  Missing | 117  27  9  2 | 75,48  17,42  5,81  1,29 | 70  21  6  2 | 72  22  6 | 47  6  3  0 | 84  11  5 |  |  |  |  |  |  |  |  |  |
| Type of complication (n=9) | Gastrointestinal tract (GIT)  Genitourinary tract (GUT)  Central nervous system (CNS)  Skin  Haematological  Neutropenia  Other  Missing | 3  3  0  6  0  1  1  1 | 20  20  0  40  0  6,67  6,67  6,67 |  |  |  |  |  |  |  |  |  |  |  |  |  |
| Radiation therapy within the last 180 days before the surgery completed | No radiation  No  Yes  Missing | 108  0  36  2 | 73,97  0  24,66  1,37 | 65  0  27  2 | 71  0  29 | 43  0  9  0 | 83  0  17 | 39,8  25,0 | **0,13** | 1  0,62 | Reference | | 0,20 | 1  0,66 | Reference | |
|  |  |  |  |  |  |  |  |  |  |  | 0,34 | 1,14 |  |  | 0,36 | 1,24 |
